# Health and reproductive experiences of women with an *FMR1* premutation with and without fragile X premature ovarian insufficiency

**DOI:** 10.3389/fgene.2014.00300

**Published:** 2014-09-08

**Authors:** Anne C. Wheeler, Melissa Raspa, Annette Green, Ellen Bishop, Carla Bann, Anne Edwards, Donald B. Bailey

**Affiliations:** RTI International, Research Triangle Park, NCUSA

**Keywords:** *FMR1* premutation, fragile X primary ovarian insufficiency, women’s health

## Abstract

Recently, research has indicated an increased risk for greater medical and emotional comorbidity and physical health symptoms among women with an *FMR1* expansion. However, these studies have generally been limited in their ability to model multiple risk factors associated with these symptoms by small numbers (*n* = 112–146) of participants. This study used survey methodology to examine the health experiences of 458 adult women with the premutation with and without a history of a fragile X primary ovarian insufficiency (FXPOI) diagnosis. Results suggest similar findings to those reported in the literature with regard to the frequency of medical, emotional, and reproductive experiences of women with the premutation. In addition to expected reproductive differences, women with a diagnosis of FXPOI were also more likely to experience dizziness, nausea, and muscle weakness than women without a diagnosis of FXPOI. Women with and without FXPOI were more likely to have used reproductive assistance and were more likely to have experienced preeclampsia during at least one pregnancy than is reported in the general population. Having comorbid depression and anxiety was predictive of increased medical conditions and increased daily physical health symptoms.

## INTRODUCTION

Expansion of repetitions of the trinucleotide sequence of cytosine and guanine (CGG) on the *FMR1* gene is the cause of several well-described disorders including fragile X syndrome (FXS), fragile X tremor ataxia syndrome (FXTAS), and fragile X primary ovarian insufficiency (FXPOI). FXS occurs in individuals who have over 200 CGG repeats (full mutation), whereas individuals with repeats between 55 and 200 are considered to have a premutation and are at increased risk of passing on an expansion to a full mutation in the next generation. FXTAS and FXPOI are associated with premutation status. In addition to these disorders, several additional medical, emotional, and cognitive challenges have been described as occurring at a greater frequency among individuals with a premutation than would be expected in the general population. These include reports of higher rates of depression, anxiety, attention challenges, hypertension, thyroid disease, fibromyalgia, and migraines (see Wheeler et al., 2014 for a review of these features).

Several studies have examined associated medical and cognitive features in individuals with a diagnosis of FXTAS as compared with those without FXTAS (Coffey et al., 2008; Hashimoto et al., 2011; Tassone et al., 2012; Yang et al., 2013), with results suggesting greater risk for those with a FXTAS diagnosis. To date, only one study (Hunter et al., 2010) has examined symptoms other than reproductive risk for women with and without a diagnosis of FXPOI. FXPOI is defined by premature ovarian failure, cessation of menses before age 40, and other indicators of early ovarian aging or dysfunction (Sullivan et al., 2011). Approximately 20% of premutation carriers will be diagnosed with FXPOI (Sherman, 2000). Even among those at younger ages who do not meet full criteria for FXPOI, women with a premutation on average experience menopause 5 years earlier than controls (Murray, 2000; Sullivan et al., 2011) and have more hot flashes (Smith et al., 2012); decreased levels of anti-Müllerian hormone (Spath et al., 2011); increased levels of follicle stimulating hormone (FSH; Murray, 2000; Hundscheid et al., 2001; Sullivan et al., 2005; Rohr et al., 2008; Welt, 2008; Spath et al., 2011); and irregular, shorter, or skipped menstrual cycles and subfertility (Allen et al., 2007; Welt, 2008). Hunter et al. (2010) also found an increased risk for depression/anxiety and thyroid problems for premutation women who had an indication of ovarian insufficiency, suggesting, as has been hypothesized in other studies (Mondul et al., 2005; Rodriguez-Revenga et al., 2008), that the increased risk for emotional challenges reported for premutation carriers may be linked to hormonal imbalances. Mid-range CGG repeats have also been associated with both increased risk for earlier menopause (Sullivan et al., 2005; Ennis et al., 2006; Allen et al., 2007; Tejada et al., 2008; Spath et al., 2011) and increased risk for depression, anxiety, and stress susceptibility (Johnston et al., 2001; Roberts et al., 2009; Hunter et al., 2012; Seltzer et al., 2012), further suggesting an association between these reproductive and emotional risks.

Although women with a premutation experience clear increased risk for reproductive challenges, only one study to date, conducted in Finland, has examined pregnancy outcomes in a small number of premutation carriers (Kallinen et al., 2000). A slightly higher risk for late pregnancy bleeding in the *FMR1* premutation women was found compared with the general population, but no other concerns were noted related to the course or outcome of pregnancy. Other than this one small study, no other studies have examined pre-, peri-, or postnatal experiences of carrier women. Further, no studies have examined rates of use of reproductive assistance among carrier women.

Several environmental factors likely play a contributing role in the relative risk for some of these reported co-morbid features. For example, Seltzer et al. (2012) found that among premutation carrier mothers with mid-range CGG repeats, those who experienced stressful life events in the previous year also experienced elevated rates of depression and anxiety. Others have suggested that environmental toxins and stressors may interact with epigenetic functions to contribute to increased risks for negative outcomes (Hagerman and Hagerman, 2013).

It is also unclear as to how much the relative stress of having a child and/or other family members with full-mutation FXS has on these more negative outcomes. Two recently conducted studies examined some features in mothers whose child has autism in contrast to mothers whose child has FXS (Smith et al., 2012; Crum-Bailey et al., 2013), with findings suggesting similar patterns of stress and emotional challenges for premutation carrier mothers of children with FXS and mothers of those with autism. Therefore, although it is likely that the *FMR1* expansion plays some role in increasing susceptibility to physical and emotional challenges, there are clearly additional environmental contributions that must be considered in determining relative risk.

Many of the studies on individuals with a premutation have generally been limited in their ability to model multiple risk factors associated with these symptoms by small numbers (*n* = 112–146) of participants. This study used survey methodology to contribute to knowledge regarding the premutation phenotype by describing health experiences of a large number of women across a wide adult age range. In addition to providing frequencies of reported symptoms over adult age groups, we also provide a comparison of these symptoms in women with a FXPOI diagnosis compared with those without an FXPOI diagnosis. Finally, we examine environmental predictors of several of these symptoms to aid in identifying possible additional risk factors for women with a premutation.

## MATERIALS AND METHODS

### DESIGN

Survey methodology was used to describe the self-reported experiences with various physical, psychological, and reproductive symptoms of adult women with an *FMR1* premutation. Survey items were based on comorbid medical and psychological diagnosis and daily physical health symptoms previously reported to occur at a greater frequency among women with a premutation (Smith et al., 2012). Participants were offered the opportunity to complete the survey online or via telephone; the majority completed the survey online.

### PARTICIPANTS

All procedures were approved by the Institutional Review Board (IRB) of the authors’ primary institutions. A total of 454 adult women with the premutation completed this survey as part of a larger national survey study on the experiences of families affected by FXS. The number of participants included in individual analyses varies slightly as a result of skipped items on the survey.

Of the women with a premutation who completed the survey, 13 (3%) self-reported a diagnosis of FXTAS, 72 (16%) self-reported a diagnosis of FXPOI, and 3 (0.006%) indicated a history of both FXTAS and FXPOI. The rate of self-reported FXPOI among this sample is similar to previously reported prevalence rates among premutation carriers (Sherman, 2000). The self-reported rate of FXTAS (3%) is lower than previously reported rates among female premutation carriers, which is to be expected given the web-based format of this study and lower likelihood of older individuals with complications inherent with FXTAS being involved with a computer-based study.

Because of the small number of women with a diagnosis of FXTAS, these participants were removed from further analyses, leaving a total *n* of 438. Frequencies of comorbid diagnoses, physical health symptoms, and reproductive experiences are reported separately for women with no history of FXPOI (no FXPOI group; *n* = 364) and those with a history of FXPOI (FXPOI group; *n* = 72).

The respondents were primarily white (94%) and well educated (67% with at least a 4-year college degree), with an average age of 48.5 years. Family income was also generally high with 41% reporting annual incomes over $100,000. Demographic information for individuals with and without a reported FXPOI diagnosis is reported in **Table 1**.

**Table 1:** Demographics of the sample with and without FXPOI.

	Women without FXPOI	Women with FXPOI
*N*	365	73
Mean age (SD)	48.63 (11.69)	48.85 (12.16)
**Race**
White	93.4% (341)	94.5% (69)
Nonwhite	6.6 (24)	5.5% (4)
**Education**
HS or less	8.0% (29)	4.1% (3)
Some college	17.9% (65)	15.1% (11)
2 year degree	8.5% (31)	6.9% (5)
4 year degree	37.4% (136)	31.5% (23)
Graduate or professional degree	28.3% (103)	42.5% (31)
**Family income**
<$25,000	5.1% (18)	5.7% (4)
$25,000–50,000	16.1% (57)	11.4% (8)
$50,001–75,000	21.2% (75)	15.7% (11)
$75,001–100,000	17.8% (63)	18.6% (13)
>$1,000,000	39.8% (141)	48.6% (34)
Number of total children*
0	2.74% (10)	6.9% (5)
1	47.1% (172)	61.6% (45)
2	34.8% (127)	21.9% (16)
3+	15.3% (56)	9.6% (7)
Number of children with full mutation or premutation FX**
0	6.6% (24)	11.3% (8)
1	68.5% (248)	78.9% (56)
2	21.6% (78)	7.1% (5)
3	3.3% (12)	2.8% (2)
Mean aggressive behavior score (SD)	5.37 (4.27)	5.25 (3.94)

*Significant difference between groups: *X*^2^ = 9.62; df = 3; *p* = 0.02.

**Significant difference between groups: *X*^2^ = 10.32; df = 3; *p* = 0.01.

### MEASURES

Survey items included questions about whether the respondent had ever been diagnosed or treated for seven specific medical [thyroid disease, hypertension, autoimmune disease, heart disease, gastrointestinal (GI) issues, seizures, diabetes] and five psychological or educational (depression, anxiety, ADHD, learning disabilities, speech/language disorder) diagnoses. They were also asked to report on the frequency of specific physical symptoms (e.g., headache, fatigue, joint pain) experienced over the previous 30 days. These symptoms were reported on a 4 point scale (0 = never to 3 = very often). For reproductive health items, women were asked about whether they had experienced absent or irregular periods, early menopause, and infertility. They were also asked about their use of reproductive assistance both before and after learning of their own *FMR1* status. Respondents indicated whether they had any challenges with pregnancy (e.g., preeclampsia), birth and delivery (e.g., length of labor), and the postpartum period [e.g., difficulty breastfeeding, postpartum depression (PPD)]. In other modules of the survey, women provided information regarding family income, education level, and number of children with and without FXS. Finally, parents reported on the frequency and severity of aggressive behavior as a measure of problem behavior in their children (with full or premutation FX). For the purpose of these analyses if there was more than one child in the family, we chose the aggressive behavior score for the child with the most challenging behaviors.

### ANALYSIS

Descriptive analyses included the frequencies of responses for women with and without FXPOI, as well as comparison tests across age groups. Regression models were run including all women with and without FXPOI to examine predictors of reproductive symptoms, number of co-occurring medical conditions, and a composite of daily physical health symptoms. Models were initially run separately for FXPOI and no-FXPOI groups, as well as examining age by diagnoses interactions with no differences in results. Therefore, we examined possible environmental predictors for increased reproductive challenges irrespective of FXPOI diagnosis (see **Table 2**). Predictor variables included age, income, child aggressive behaviors, and presence of depression and/or anxiety (emotional composite).

**Table 2:** Regression models of health and reproductive outcomes.

Variable	Medical Conditions		Daily Health Symptoms		Reproductive	
	B (SE)	*p*	B (SE)	*p*	B (SE)	*p*
**Age**
≤40	REF		REF		REF	
41–50	0.37 (0.15)	0.014	0.27 (0.13)	0.430	0.27 (0.13)	0.043
51–60	0.58 (0.17)	<0.001	0.02 (0.15)	0.744	0.02 (0.15)	0.901
≥61	1.14 (0.19)	<0.001	0.10 (0.17)	0.947	0.10 (0.17)	0.561
**Income**
≤$50,000	REF		REF		REF	
$50,001–$75,000	-0.31 (0.17)	0.079	-0.12 (0.16)	0.014	-0.12 (0.16)	0.461
$75,001–$100,000	-0.33 (0.17)	0.056	0.07 (0.16)	0.003	0.07 (0.16)	0.645
>$100,000	-0.57 (0.15)	<0.001	-0.03 (0.14)	<0.001	-0.03 (0.14)	0.812
**Child Aggression score**
≤2	REF		REF		REF	
4	-0.01 (0.16)	0.936	0.16 (0.14)	0.607	0.16 (0.14)	0.273
7	0.08 (0.15)	0.581	0.09 (0.14)	0.046	0.09 (0.14)	0.503
≥8	0.11 (0.16)	0.490	0.19 (0.14)	0.053	0.19 (0.14)	0.190
**Emotional conditions**
0	REF		REF		REF	
1	1.25 (0.14)	<0.001	-0.18 (0.13)	0.050	-0.18 (0.13)	0.164
2	2.43 (0.13)	<0.001	0.04 (0.12)	<0.001	0.04 (0.12)	0.757

*REF = reference category.*

## RESULTS

There were no differences in age, maternal education, race, family income, number of children with FXS and comorbid autism, and child aggressive behaviors between the FXPOI and no FXPOI groups. Women with a diagnosis of FXPOI had fewer total children (*X*^2^ = 9.62; df = 3; *p* = 0.02) and fewer children with full-mutation or premutation FX (*X*^2^ = 10.32; df = 3; *p* = 0.02) than women without a diagnosis of FXPOI (see **Table 1**).

### COMORBID PSYCHIATRIC/EDUCATIONAL DIAGNOSES

**FIGURE 1:**
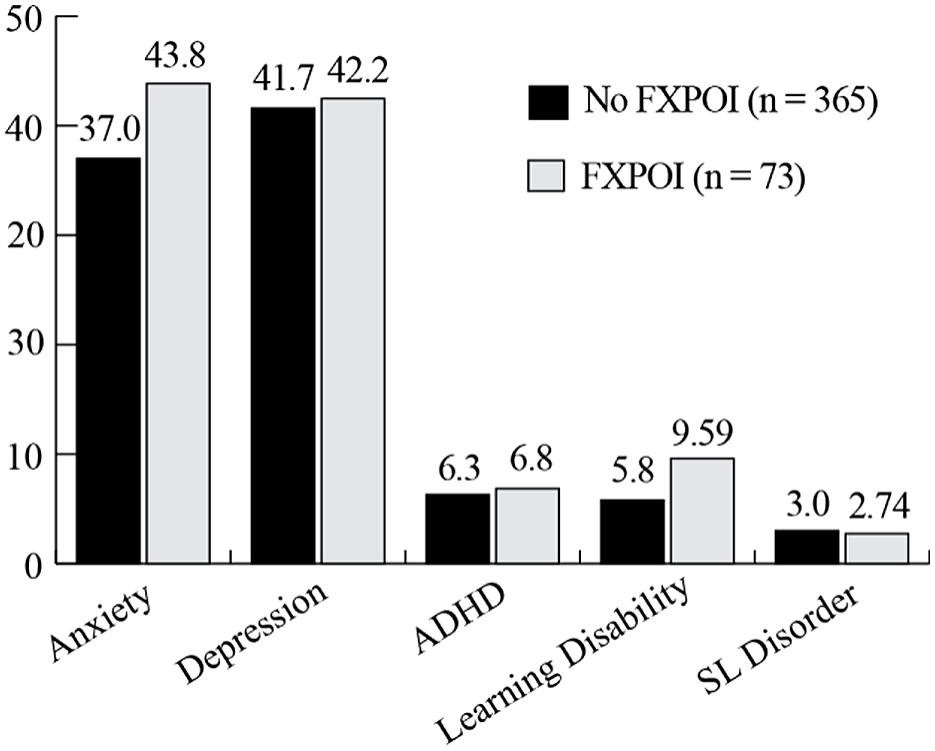
**Percentage of women with and without FXPOI endorsing co-occurring psychiatric or educational conditions**.

**FIGURE 2:**
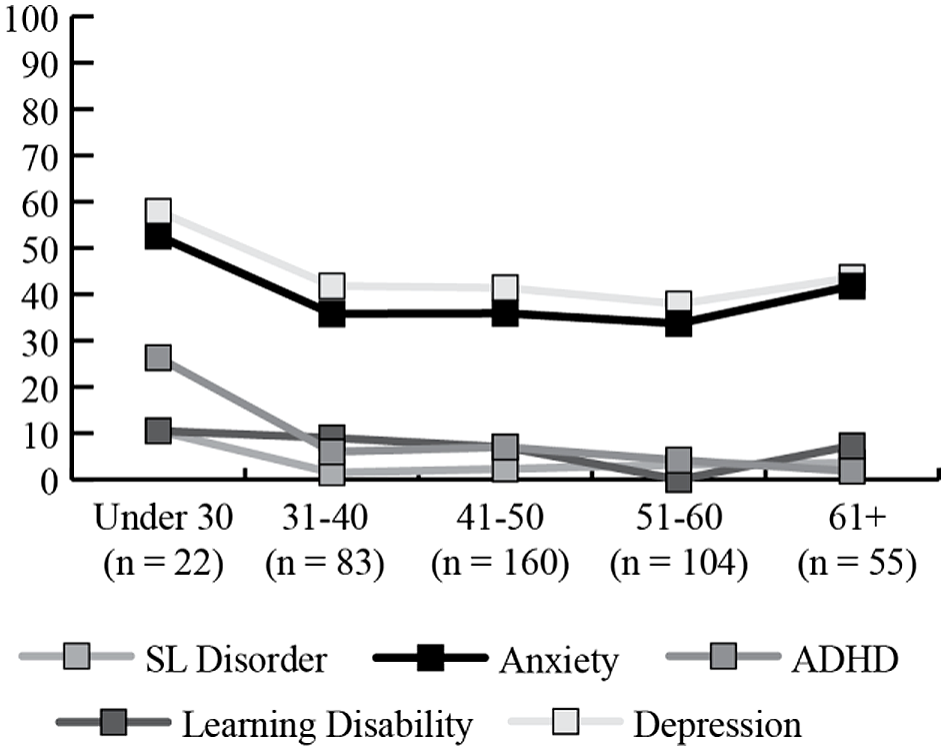
**Percentage of women endorsing co-occurring psychiatric or educational conditions, by age group**.

Depression and anxiety were both reported at high rates with around 40% of women with and without FXPOI reporting a history of these diagnoses. Across all age groups over a third of women reported a history of depression and/or anxiety with a slightly higher (but not significant) percentage of women under 30 and over 61 reporting these diagnoses. No significant differences were found for incidence of any psychiatric or educational diagnoses between those with and without FXPOI. No differences across age groups were found. See **Figure 1** and **Figure 2** for the percentage of women endorsing psychiatric or educational diagnoses.

### COMORBID MEDICAL DIAGNOSES

**FIGURE 3:**
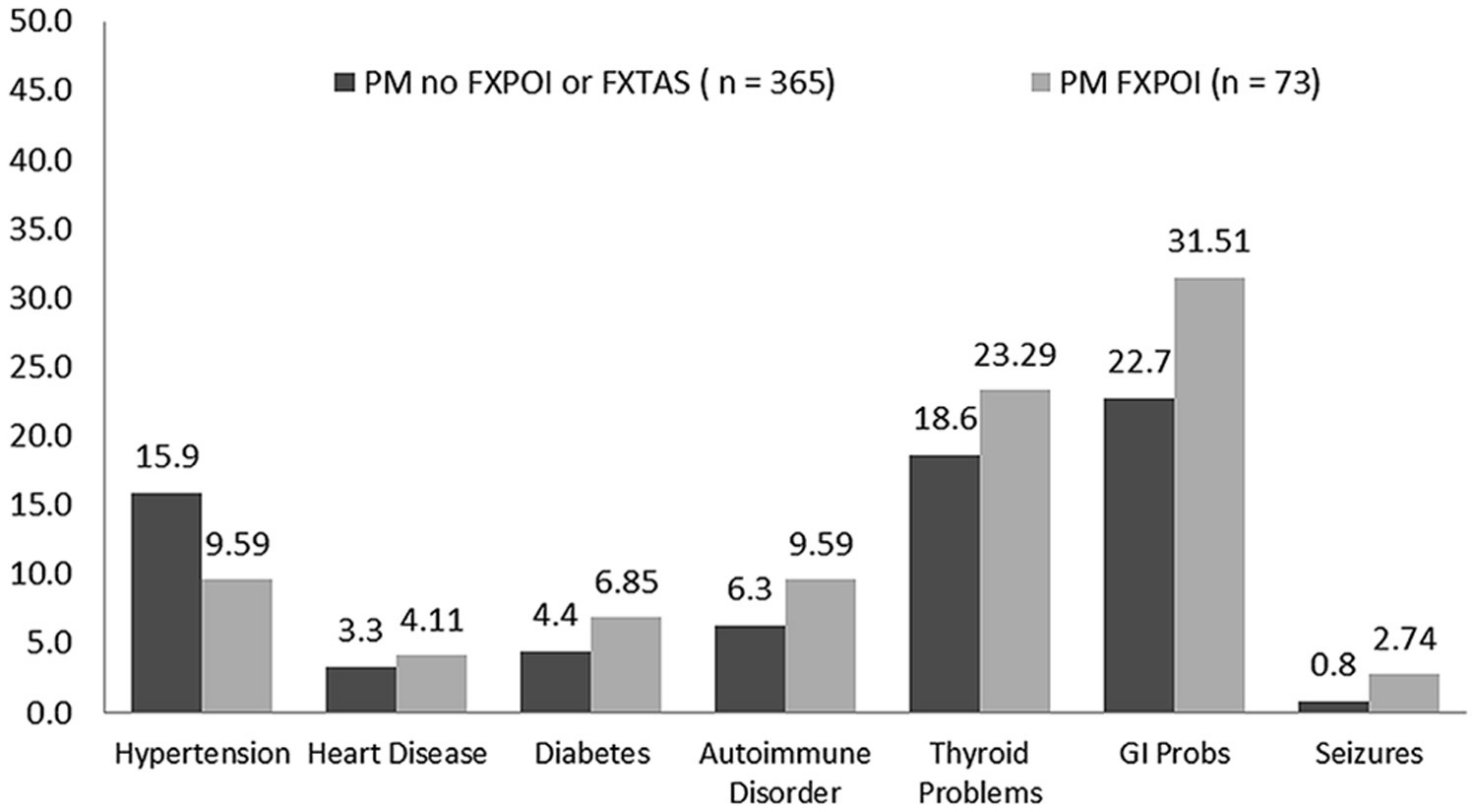
**Percentage of women with and without FXPOI endorsing co-occurring medical conditions**.

**FIGURE 4:**
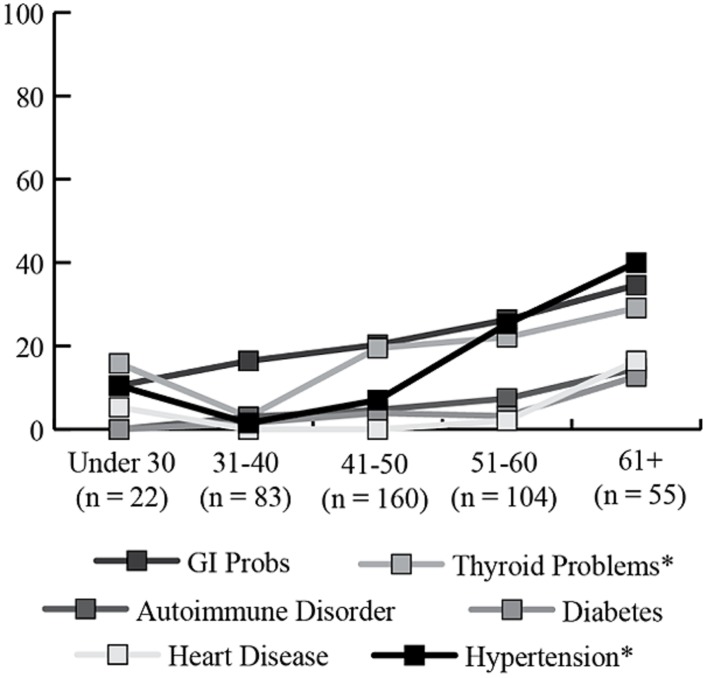
**Percentage of women endorsing co-occurring medical conditions, by age group.** *Significant increases in older age groups.

The percentage of women with a diagnostic history of the seven medical conditions is reported separately for women with and without a diagnosis of FXPOI in **Figure 3**. A little under a quarter of women with (23.3%) and without (18.6%) FXPOI reported a history of thyroid disorder, and nearly a third with (31.5%) and without (22.7%) FXPOI reported a history of GI issues. There were no significant differences found between women with and without a diagnosis of FXPOI on any of the medical diagnoses. All medical conditions were reported at higher rates by older adults (see **Figure 4**), with a significant increase over age groups for hypertension (*X*^2^ = 49.96; df = 4; *p* < 0.001) and thyroid disease (*X*^2^ = 19.86; df = 4; *p* < 0.001).

### DAILY PHYSICAL HEALTH SYMPTOMS

**FIGURE 5:**
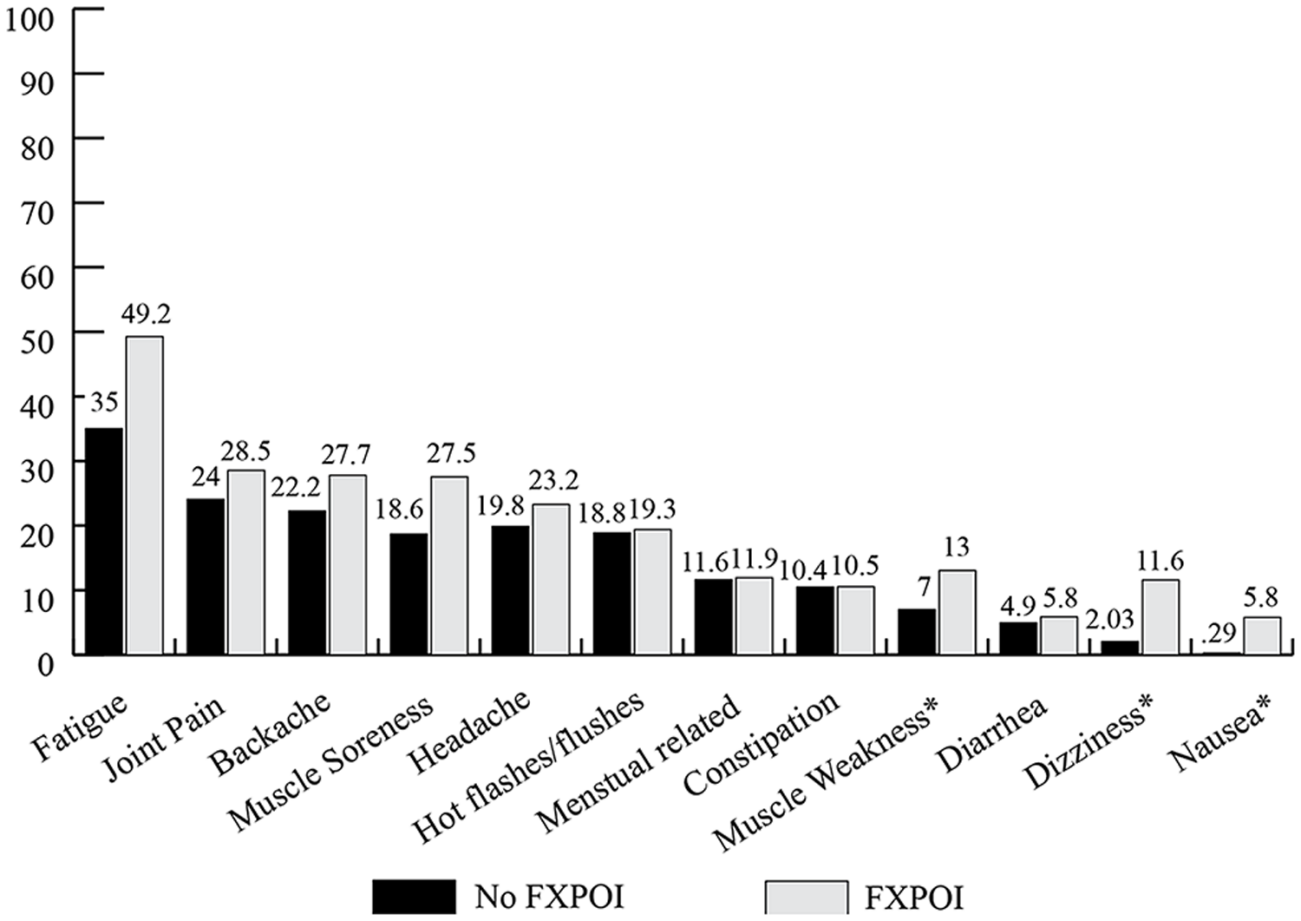
**Percentage of women with and without FXPOI endorsing having experienced specific physical health symptoms “often or very often” in the previous 30 days.** *Significant differences between groups.

Fatigue was the most commonly endorsed daily health symptom with 35% of women without FXPOI and nearly half (49.3%) of women with FXPOI reporting experiencing fatigue often or very often in the previous month (see **Figure 5**). Joint pain, muscle pain, backache, and headaches were also endorsed as occurring often or very often in around 20% of women. Women with a diagnosis of FXPOI were significantly more likely to experience muscle weakness (*X*^2^ = 8.84; df = 3; *p* = 0.03), dizziness (*X*^2^ = 12.25; df = 3; *p* = 0.007), and nausea (*X*^2^ = 8.79; df = 3; *p* = 0.01) than women without a diagnosis of FXPOI.

**FIGURE 6:**
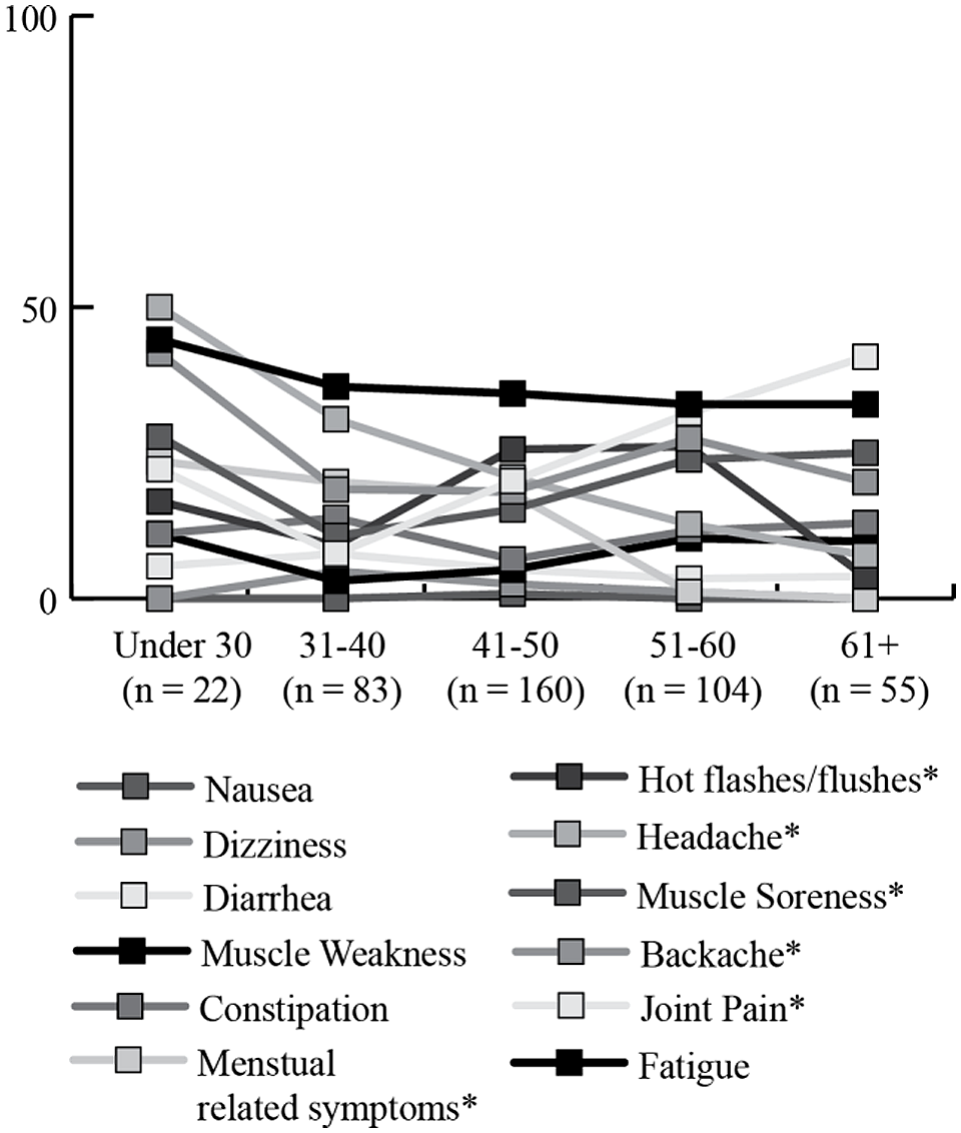
**Percentage of women endorsing having experienced specific physical health symptoms “often or very often” in the previous 30 days, by age group.** *Significant differences across age groups.

Significant age-related differences were found for headaches (*X*^2^ = 23.36; df = 4; *p* < 0.001), joint pain (*X*^2^ = 22.28; df = 4; *p* < 0.001), menstrual symptoms (*X*^2^ = 27.72; df = 4; *p* < 0.001), and hot flashes (*X*^2^ = 18.85; df = 4; *p* < 0.001). Headaches, menstrual symptoms, and hot flashes decreased in the older age groups, while joint pain tended to be endorsed more by older women (see **Figure 6**).

Results of the regression model showed that respondents with lower family income, a child with more aggression issues, and more co-occurring emotional conditions reported more daily physical health symptoms (see **Table 2**).

### REPRODUCTIVE CHALLENGES

**FIGURE 7:**
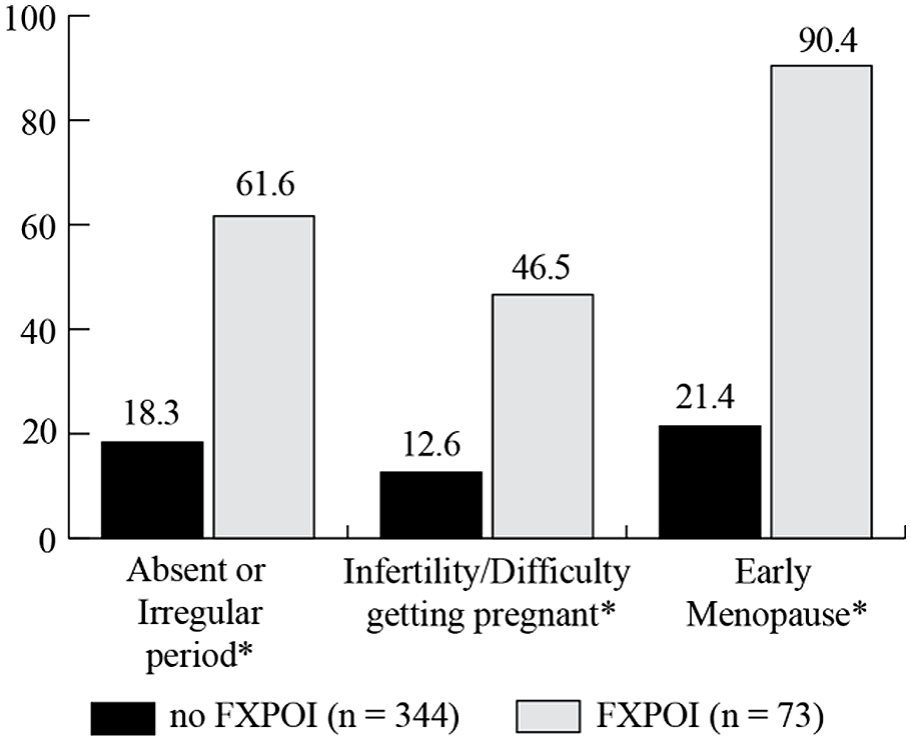
**Percentage of women with and without FXPOI experiencing reproductive challenges.** *Significant differences between groups.

A similar percentage of women with and without FXPOI reported menstrual-related daily health symptoms that occurred often or very often. However, significantly more women with FXPOI reported never experiencing daily symptoms related to menstrual issues (*X*^2^ = 13.69; df = 3; *p* ≤ 0.003), which likely reflects the women in the FXPOI group no longer menstruating at the time of the survey. Women with FXPOI, as a reflection of their diagnosis, reported high rates of irregular or absent periods (61.6%) early menopause (90.4%), and difficulty getting pregnant (46.6%), significantly higher than women without FXPOI (see **Figure 7**). However, approximately 20% of women without FXPOI also reported irregular or absent periods (18.4%), and early menopause (21.4%), and 12.6% also had difficulty getting pregnant.

Although clearly women with FXPOI experience high rates of these reproductive challenges, a significant percentage of women without a reported FXPOI diagnosis also endorsed these challenges. The only moderate predictor for reproductive challenges was age, with women between 41 and 50 reporting having ever experienced reproductive challenges more than those under 40.

Regression models indicate that age and co-occurring depression and/or anxiety were significant predictors of more comorbid medical conditions. Those with family incomes over $100,000 reported experiencing fewer medical conditions (see **Table 2**).

### USE OF REPRODUCTIVE ASSISTANCE

**FIGURE 8:**
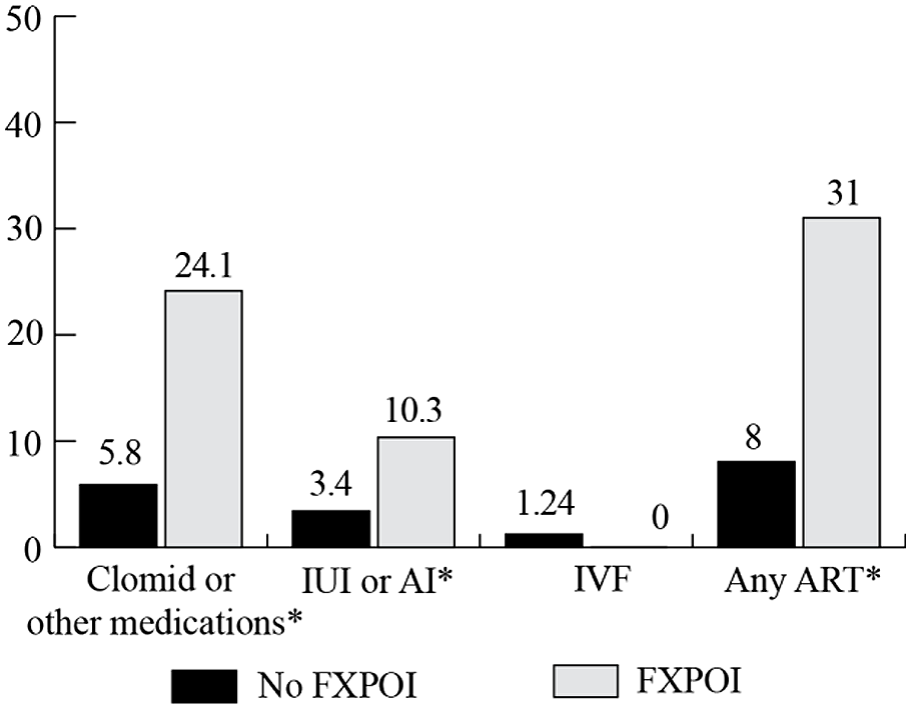
Percentage of women with and without FXPOI endorsing use of reproductive assistance *before* knowing their *FMR1* status. *Significant difference between groups.

**FIGURE 9:**
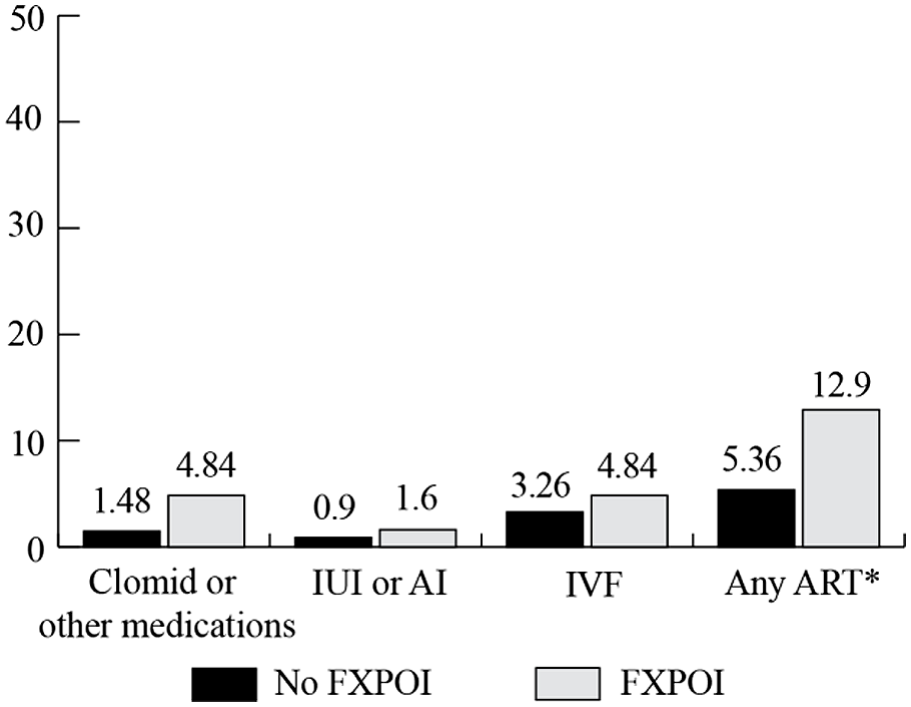
**Percentage of women with and without FXPOI endorsing use of reproductive assistance *after* knowing their *FMR1* status.** *Significant difference between groups.

Women were asked to report on the use of reproductive assistance before and after they knew about their *FMR1* premutation status (see **Figure 8** and **Figure 9**, respectively). Among the women with FXPOI, 31.1% reported having used some form of reproductive assistance before knowing their *FMR1* status, and 12.9% reported using reproductive assistance after knowing their *FMR1* status. For women without a diagnosis of FXPOI, 8.5% reported using reproductive assistance before and 5.2% reported using reproductive assistance after finding out their *FMR1* status.

Before knowing their status, fertility drugs (e.g., Clomid) were the most commonly reported reproductive assistance used by women without FXPOI (5.8%), followed by 3.4% of women reporting use of intrauterine insemination (IUI) or artificial insemination (AI) and 1.3% reported use of *in vitro* fertilization (IVF). After knowing their status, 3.5% of women without FXPOI reported using IVF, 1.5% used fertility drugs, and less than 1% used AI or IUI.

Among women with FXPOI, the most frequently reported reproductive assistance used was fertility drugs (e.g., Clomid; 24.4% before and 4.8% after knowing their *FMR1* status). IUI or AI was used by 10.3% of women with FXPOI, and none used IVF before knowing their *FMR1* status. After knowing their status, 4.8% of women reported using IVF, 4.8% used fertility drugs, and 1.6% used AI or IUI.

Women with FXPOI were significantly more likely to have used any reproductive assistance (*X*^2^ = 27.89; df = 1; *p* < 0.001), to have used fertility drugs (*X*^2^ = 17.69; df = 1; *p* ≤ 0.001), and to have used IUI or AI (*X*^2^ = 5.02; df = 1; *p* = 0.03) before knowing their status than women without FXPOI. After knowing their status, women with FXPOI were more likely to have used any reproductive assistance than women without FXPOI (*X*^2^ = 4.14; df = 1; *p* = 0.04).

No women with or without FXPOI reported using pre-implantation genetic diagnoses (PGD) before knowing their status; after knowing their status, 4.8% of women with FXPOI and 2.4% of women without FXPOI reported using PGD.

### PRE-, PERI-, AND POSTPARTUM EXPERIENCES

**FIGURE 10:**
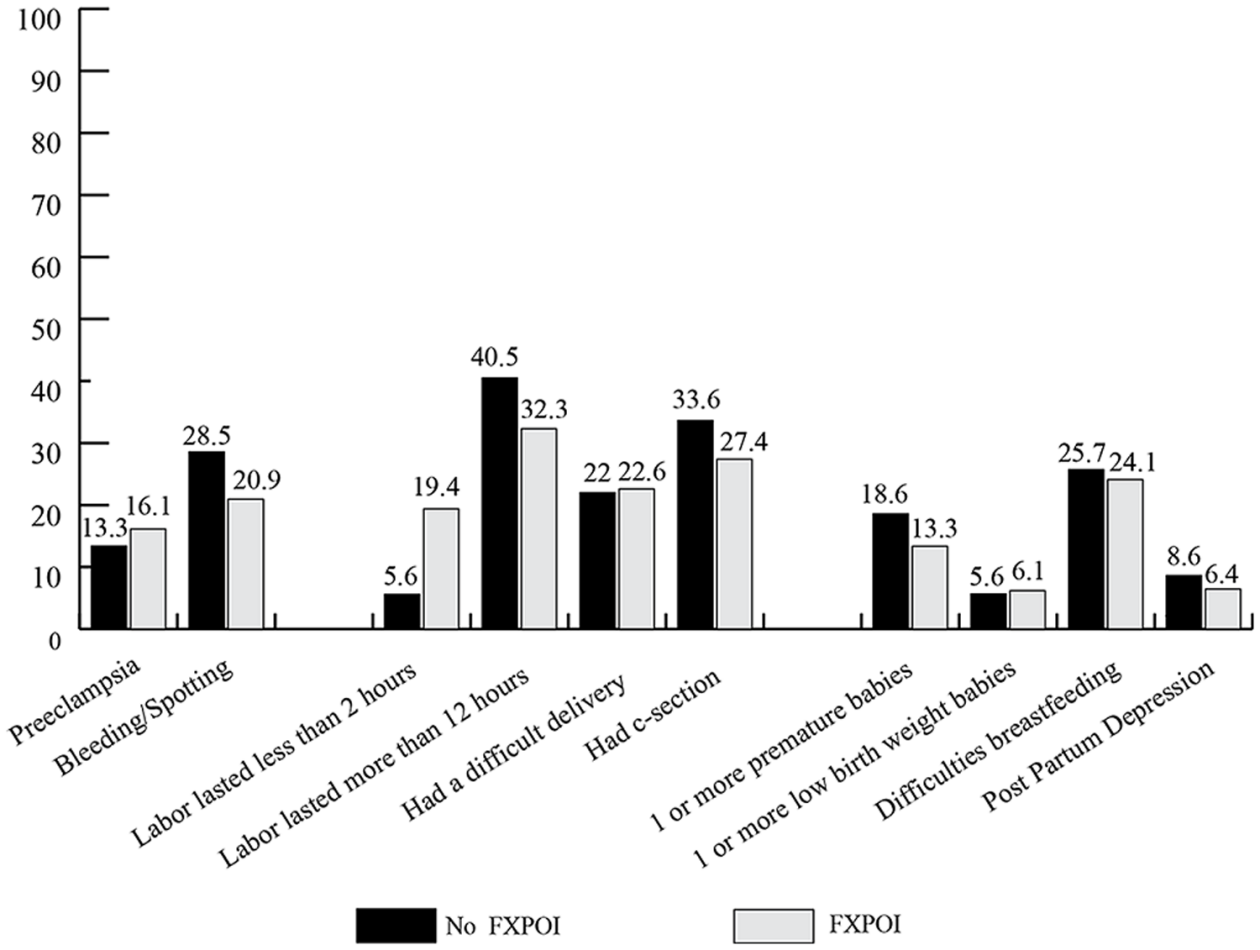
**FIGURE 10. Percentage of women experiencing pre-, peri-, or postpartum issues**.

Women were asked about specific experiences with each of their pregnancies, labor, and delivery (see **Figure 10**): 13.4% of women without FXPOI and 16.1% of women with FXPOI reported experiencing preeclampsia or high blood pressure during at least one pregnancy. About 28% of women without FXPOI and 21% of women with FXPOI reported bleeding or spotting during at least one pregnancy.

With regard to labor and delivery, about 22% of women with and without FXPOI reported a difficult delivery with at least one child. Around a third of women in both groups reported having had a Caesarean section, and for 40.5% of women without FXPOI and 32.3% of women with FXPOI, labor lasted over 12 h. Women with FXPOI reported precipitous labor (less than 2 h) at a significantly higher rate than women without FXPOI (*X*^2^ = 12.71; df = 1; *p* = 0.004).

Less than 20% (18.6%) of babies born to women without FXPOI were born before 37 weeks gestation, while 13.35% of babies born to women with FXPOI were born premature. These rates are similar to what is reported in the general population. Similarly, around 6% of babies born to women with or without FXPOI were born with a low birth weight. Approximately one-quarter of women with and without FXPOI who reported having breastfed for at least 1 week endorsed stopping primarily because of difficulties for her (as opposed to difficulties for the child).

Less than 10% of women with and without FXPOI endorsed having experienced PPD. Of those who reported experiencing PPD, 25% of women with FXPOI and 43.3% of women without FXPOI endorsed having experienced it two or more times. Of the 19 women total who experienced one episode of PPD, roughly a third (6, 32%) reported a short duration of less than 6 weeks, a third (7, 37%) reported a duration of 6 weeks to 6 months, and a third (6, 32%) reported a duration of over 6 months. Of the 14 women total who reported experiencing multiple cases of PPD, half reported experiencing at least one PPD episode lasting over 6 months. There were no differences between those with and without FXPOI on occurrence (*X*^2^ = 0.47; df = 1; *p* = 0.49) or duration (*X*^2^ = 0.08; df = 1; *p* = 0.77) of PDD.

## DISCUSSION

Results from this large survey study expand upon previous findings suggesting increased health risks for women with an *FMR1* premutation. In addition, this study examined rates of comorbid conditions, daily physical health symptoms, and reproductive issues (including prenatal and postpartum experiences) of women with and without a diagnosis of FXPOI. Further, this study examined environmental predictors of health and reproductive issues experienced by women with the premutation.

### CO-OCCURRING MEDICAL AND PSYCHIATRIC CONDITIONS

There were no significant differences between women with and without FXPOI on any of the comorbid medical or psychiatric/educational conditions. As previously reported, high rates of depression and anxiety were endorsed in this sample, with consistent frequencies reported across the adult age groups. Increased risks for depression and anxiety have been described as being related to genetic risk associated with an *FMR1* expansion (Johnston et al., 2001; Hunter et al., 2008; Roberts et al., 2009), with some reports noting increased risks for carrier women prior to having a child with FXS (Roberts et al., 2009). However, other studies have noted that rates of depression and anxiety among carrier women do not differ significantly from mothers of children with autism (Smith et al., 2012). Higher rates of depression and anxiety have also been reported among women with more reproductive symptoms (Hunter et al., 2010), although in this study we did not find differences in rates of these psychiatric conditions between those with and without FXPOI. More studies are needed to identify risks for depression and anxiety related to genetic versus environmental factors in women with a premutation.

The most commonly reported co-occurring medical conditions for those with and without FXPOI were GI problems, thyroid problems, and hypertension. Rates of heart disease, autoimmune disorders (including fibromyalgia), diabetes, and seizures were all under 10% for participants in this sample, which are consistent with national occurrence rates (Blackwell et al., 2014). As would be expected, rates of all comorbid medical concerns, except for seizures and GI problems, were higher for the older women.

Hypertension has been noted to occur at high rates among premutation carriers with FXTAS (Coffey et al., 2008). Among those without FXTAS, however, the risk was no different than controls. Similarly, in this study, rates of hypertension were not higher than reported in national occurrence rates (Blackwell et al., 2014) and, as in the general population, rates increased with age. Among non-FXTAS carriers, therefore, there does not appear to be a greater risk for hypertension than would be expected in the general adult population. A potential increased risk for preeclampsia (discussed further below), however, may prove to be an important variable in determining who may be at greater risk for hypertension in later adulthood.

Similarly, around a quarter of all women in this study reported a history of thyroid disease. This rate is similar to previous reports of thyroid disease in carrier women without FXTAS, which has previously been reported to be slightly, but not significantly, higher than control samples (Coffey et al., 2008; Hunter et al., 2010). Younger women in this study did report rates somewhat higher than has been reported in national occurrence rates [11 vs. 3% (Blackwell et al., 2014)]. However, rates were more similar to what would be expected in the general population for older adults. As in the general population and previous studies of premutation carriers, rates increased with age, and age was the only significant predictor of thyroid problems in this sample. Whereas it has previously been reported that women with FXPOI may be at greater risk for thyroid problems than those without FXPOI (Hunter et al., 2010), we did not find significant differences between the groups. Additional studies are needed to examine specific genetic biomarkers, especially in younger women, which may predict increased risk for thyroid problems.

GI problems were also reported at relatively high rates, with nearly a third of women with and without FXPOI endorsing these issues. Further, of all of the medical co-occurring conditions, GI problems were one of the only ones that did not increase across age groups, suggesting these issues are pervasive across the adult life span. Unfortunately, the lack of specificity of this item did not allow for direct comparisons with national occurrence rates. However, Smith et al. (2012) found that carrier mothers of children with FXS reported significantly more GI problems than mothers of children without disabilities but not more than mothers of children with autism. This would suggest that GI problems may be related to stress and anxiety associated with caregiving, and indeed, GI problems have been linked with increased stress in the general population (Mayer, 2000). More research exploring this health concern is needed.

### DAILY HEALTH SYMPTOMS

Fatigue, joint pain, backache, muscle soreness, and headaches were the most commonly reported daily health symptoms by women with and without FXPOI. These same symptoms were also the most commonly reported by Smith et al. (2012) in a daily diary study where carrier mothers of children with FXS reported symptoms experienced that day via a telephone interview each night for eight consecutive days. All of these symptoms were reported at a greater frequency than mothers of children without disabilities. The consistency in reports of these symptoms suggests these issues are important aspects’ affecting the daily lives of *FMR1* premutation mothers of children with FXS. In this sample, child problem behaviors were predictive of daily health symptoms. Similarly, in the Smith et al. study, these symptoms were experienced as much or more by mothers of children with autism, suggesting the increased rate of these health challenges may be due more to stressful caregiving than to the *FMR1* premutation per se. Comorbid emotional conditions (depression and/or anxiety) were strongly predictive of daily physical health symptoms, further suggesting stress may be playing a major role in these symptoms. Experiencing daily pain and fatigue is certainly likely to affect one’s quality of life and ability to effectively parent and, therefore, is an important consideration both clinically and for future research.

Fatigue is an area deserving additional focus for premutation mothers. Fatigue was a consistently reported symptom for nearly half of all participants across all age groups and was reported at similar rates among women with and without FXPOI. Further, sleep issues, including restless leg syndrome and sleep apnea, have been reported as occurring at high rates among premutation carriers with and without FXTAS (Hamlin et al., 2011; Summers et al., 2014). Fatigue has been associated with higher levels of stress and depression and lower levels of parenting satisfaction and efficacy in mothers of children with autism (Giallo et al., 2013). It is also associated with reduced daily functioning, clarity of thinking (Fisher et al., 2004), and ability to cope with daily stressors (Ward and Giallo, 2008; Giallo et al., 2013). The primary factors contributing to fatigue and the impact it has on daily quality of life and parent–child interactions is an important factor for future research in this population.

Women with FXPOI were significantly more likely to experience dizziness, nausea, and muscle weakness than women without FXPOI. Given the only environmental difference we examined between the two groups was the total number of children and number of children with FX, these differences in symptoms are most likely due to a biological factor, such as changes in estrogen levels associated with menopause. However, they could possibly also be related to increased risks as a result of mid-range CGG repeats or other molecular functions. More research is needed with regard to genetic and environmental risks for physical health symptoms experienced by carrier women.

### REPRODUCTIVE HEALTH

Results related to reproductive health were largely unsurprising and confirm previously reported findings suggesting elevated rates of reproductive risk for women with an *FMR1* premutation. Consistent with the hallmark symptoms of FXPOI, women with a FXPOI diagnosis reported high rates of reproductive challenges including irregular or absent periods, early menopause, and difficulty getting pregnant. However, around 20% of women without FXPOI reported these same symptoms, which was higher than reports of infertility issues among the general population (∼6%; Blackwell et al., 2014). As has been previously suggested (Hagerman and Hagerman, 2013), these symptoms likely reflect a spectrum of involvement for premutation carriers, rather than a clear designation of diagnostic threshold. Women with FXPOI were significantly more likely to have used reproductive assistance before and after knowing their *FMR1* status. Across all women with a premutation in this study, the only significant environmental predictor of reproductive challenges was age, with an expected association of women in the typical menopausal age range (41–50) reporting having experienced more challenges than younger women. These findings suggest that environmental factors, such as income, stressful child behaviors, or increased emotional challenges are not likely to increase risk for reproductive issues over and beyond risk associated with the molecular changes in *FMR1* for these women.

Most experiences with pregnancy, birth, and labor were consistent with numbers reported in the general population of women, suggesting that despite increased challenges with getting pregnant, there are generally no greater prenatal, neonatal, or postpartum risks for women with an *FMR1* premutation. One exception to this was preeclampsia, which was reported to have occurred at rates more than double what has been reported for the general population (Ananth et al., 2013). Preeclampsia is considered a multi-systemic syndrome characterized by hypertension during pregnancy (Trogstad et al., 2011). In addition to increased negative risks for the fetus and for poorer birth outcomes, preeclampsia also increases risk of maternal death in severe cases (Lain and Roberts, 2002). Preeclampsia has also been suggested as a possible risk factor for future cardiovascular issues in women in the general population (Trogstad et al., 2011). No clear factors have been associated with increased risk for preeclampsia, although maternal genetic variables, environmental exposures, and paternal contributions have been suggested (Trogstad et al., 2011). Given previous conflicting reports of higher rates of general hypertension among premutation carriers, this increased rate of preeclampsia should be explored further in future studies.

### LIMITATION AND FUTURE DIRECTIONS

This study has some important limitations to consider. First, it relied completely on maternal report and memory of experiences, which may have occurred several decades in the past (e.g., experiences with pregnancy and labor for women in their 60s). The sample was non-representative, with primarily white, well-educated women responding. Given income was a significant predictor for daily health symptoms, possible health disparities as a function of socioeconomic status are an important factor to consider in future studies.

We were also not able to get confirmation of genetic status and do not have independent reports from medical professions of the endorsed co-occurring conditions. Given several studies have shown an association between CGG repeats and risks for FXPOI and other co-occurring conditions, our lack of more detailed genetic data limits our ability to model risks for these women. Further, all of the women in this study were parents or family members of one or more individuals with FXS; therefore, we are unable to separate the impact of stressful caregiving from the effect of the *FMR1* premutation. Although for some conditions we were able to compare the results to national occurrence rates, the lack of a control group limits the ability to make conclusions regarding the relative health impact for premutation carriers.

However, despite these limitations, this study provides further suggestion of increased risks for medical and psychiatric conditions for women with a premutation, as well as the daily health challenges experienced by premutation carrier mothers. Further, it expands on studies examining differences between individuals with and without a diagnosed fragile X associated disorder and the possible association between emotional conditions and medical diagnoses and daily health symptoms. It is also the first study to report a possible increased risk for preeclampsia among carrier women. Additional research is needed to further understand the prevalence and severity of health features associated with the premutation as well as the genetic and environmental factors that contribute to the likelihood that women with a premutation will experience these additional challenges.

## Conflict of Interest Statement

The authors declare that the research was conducted in the absence of any commercial or financial relationships that could be construed as a potential conflict of interest.
